# Are 100 enough? Inferring acanthomorph teleost phylogeny using Anchored Hybrid Enrichment

**DOI:** 10.1186/s12862-015-0415-0

**Published:** 2015-06-14

**Authors:** Ron I. Eytan, Benjamin R. Evans, Alex Dornburg, Alan R. Lemmon, Emily Moriarty Lemmon, Peter C. Wainwright, Thomas J. Near

**Affiliations:** Department of Ecology & Evolutionary Biology and Peabody Museum of Natural History, Yale University, New Haven, 06520 CT USA; Department of Marine Biology, Texas A&M University at Galveston, Galveston, 77553 TX USA; Department of Scientific Computing, Florida State University, Dirac Science Library, Tallahassee, 32306 FL USA; Department of Biological Science, Florida State University, Biomedical Research Facility, Tallahassee, 32306 FL USA; Department of Evolution & Ecology, University of California, One Shields Avenue, Davis, 95616 CA USA

**Keywords:** Ovalentaria, Anchored hybrid enrichment, Phylogenomics, Cichlidae, Blenniiformes, Acanthomorpha, Percomorpha, *Pholidichthys*

## Abstract

**Background:**

The past decade has witnessed remarkable progress towards resolution of the Tree of Life. However, despite the increased use of genomic scale datasets, some phylogenetic relationships remain difficult to resolve. Here we employ anchored phylogenomics to capture 107 nuclear loci in 29 species of acanthomorph teleost fishes, with 25 of these species sampled from the recently delimited clade Ovalentaria. Previous studies employing multilocus nuclear exon datasets have not been able to resolve the nodes at the base of the Ovalentaria tree with confidence. Here we test whether a phylogenomic approach will provide better support for these nodes, and if not, why this may be.

**Results:**

After using a novel method to account for paralogous loci, we estimated phylogenies with maximum likelihood and species tree methods using DNA sequence alignments of over 80,000 base pairs. Several key relationships within Ovalentaria are well resolved, including 1) the sister taxon relationship between Cichlidae and *Pholidichthys*, 2) a clade containing blennies, grammas, clingfishes, and jawfishes, and 3) monophyly of Atherinomorpha (topminnows, flyingfishes, and silversides). However, many nodes in the phylogeny associated with the early diversification of Ovalentaria are poorly resolved in several analyses. Through the use of rarefaction curves we show that limited phylogenetic resolution among the earliest nodes in the Ovalentaria phylogeny does not appear to be due to a deficiency of data, as average global node support ceases to increase when only 1/3rd of the sampled loci are used in analyses. Instead this lack of resolution may be driven by model misspecification as a Bayesian mixed model analysis of the amino acid dataset provided good support for parts of the base of the Ovalentaria tree.

**Conclusions:**

Although it does not appear that the limited phylogenetic resolution among the earliest nodes in the Ovalentaria phylogeny is due to a deficiency of data, it may be that both stochastic and systematic error resulting from model misspecification play a role in the poor resolution at the base of the Ovalentaria tree as a Bayesian approach was able to resolve some of the deeper nodes, where the other methods failed.

**Electronic supplementary material:**

The online version of this article (doi:10.1186/s12862-015-0415-0) contains supplementary material, which is available to authorized users.

## Background

Assembling the tree of life is one of the primary goals of systematic biology [1]. There is substantial progress towards the resolution of major lineages of vertebrates including birds [2], mammals [3], squamates [4, 5], and ray-finned fishes [6–8]. However, most phylogenetic studies with comprehensive taxon sampling that use large DNA sequence datasets continue to exhibit several shallow and deep nodes in the phylogeny that remain poorly resolved [9–11]. It is generally not clear if the lack of resolution at a particular node in a phylogenetic tree is the result of random and systematic estimation error [12], incomplete lineage sorting exacerbated by relatively rapid lineage diversification [13], or the lack of phylogenetic signal to resolve short internodes in phylogenetic trees [14, 15]. Increased sampling of DNA sequence data may help resolve poorly supported nodes when lack of resolution is driven by ancestral polymorphism and limited phylogenetic signal; however, conclusions from simulations and empirical studies are equivocal [16–20].

The advent of high-throughput sequencing technology offers a strategy to rapidly collect large amounts of data for phylogenetic inference [21, 22]. Phylogenomic datasets provide resolution to both shallow [16] and deep [23] phylogenetic relationships by employing different classes of markers, dependent on the time scale of divergence among the lineages in a particular study (see [24] for a review). This flexibility in phylogenomic data sampling strategies allows investigators to collect DNA sequence data that facilitates the simultaneous resolution of both shallow and deep phylogenetic divergences.

Hybrid enrichment, or sequence capture, uses short DNA sequences as capture probes that are designed to target areas of interest in a genome. Once these targets are captured they are sequenced using next-generation methods [25]. These probes can be designed for any part of the genome under study, whether for targeting loci associated with human diseases [26] or specific genes for phylogenetic inference [21, 22]. At least two different hybrid enrichment methods are currently being used for phylogenomics, each targeting different regions of the genome. The ultraconserved element approach (UCE) targets very highly conserved regions of the genome to capture non-coding regions of the genome. The UCEs Faircloth et al. [21] used in their initial probe set were identified in the genomes of two birds and one lizard, making an amniote-oriented kit. The anchored hybrid enrichment method (AHE) instead targets a set of loci that are primarily in coding regions of the genome. In AHE, probes are designed specifically to highly conserved and widely distributed regions of the genome that are flanked by less conserved regions. The loci used in the AHE kit were identified using broader and deeper taxonomic sampling than what was used for the original UCE design, increasing capture efficiency for a wider taxonomic range relative to those markers. This facilitates the capture of homologous loci that are useful for both old and more recent divergences, a property shared with UCEs [22, 27].

One advantage to AHE, which we utilize here, is the ease of generating reliable alignments due to the paucity of gapped regions and saturated sites in the target regions. Another advantage is increased levels of phylogenetic information in target regions, compared to those targeted in UCE studies (A.R. Lemmon, unpub. data), as a consequence of targeting more variable regions of the genome. The original AHE probe set was designed by comparing the genomes of five vertebrate lineages: humans, squamates, birds, amphibians, and teleost fishes. This provided 512 coding genes for phylogenetic inference [22]. While this strategy provided a broad taxonomic focus, the species used in this first probe set were not necessarily ideal as model taxa. For instance, the fish species used, *Danio rerio*, is over 250 million years divergent from a large proportion of teleost fishes [6]. Thus, the first iteration of the vertebrate kit may be expected to be only partially successful in capturing the full suite of loci because of the large divergence between the model species and the experimental ones.

An advantage of hybrid enrichment is that it allows the capture of all the homologues of a gene. However, this gives rise to the uncertainty as to whether the sequences aligned in a phylogenetic matrix are orthologous. This is of particular concern in teleost fishes, where there has been a whole genome duplication event (WGD) prior to the diversification of all living teleosts [28, 29]. An alignment with paralogous genes could produce a gene-family tree, not the true species tree, or the differential loss of duplicate gene copies could lead to discordance between gene trees and species trees [30]. In addition, if multiple copies of paralogous loci are used to represent one individual’s sequence, such as through generating consensus sequences across gene copies, this would lead to false phylogenetic signal [31]. This situation could be especially problematic when a majority of the sampled lineages are represented with only one individual and the true species tree is generally unknown. The predicted result of non-orthologous loci in a dataset is the inference of an inaccurate phylogeny, especially in situations where there is weak phylogenetic signal in a dataset [31, 32]. Thus, accurate assessment of orthology is essential in teleost phylogenomics studies.

Ovalentaria is a clade of teleost fishes containing more than 4800 species that are classified into 40 taxonomic families. This lineage comprises more than 27 % of all percomorph teleosts and approximately 16 % of all living ray-finned fishes [33]. Relaxed molecular clock analyses estimate the age of Ovalentaria at approximately 91 million years (Ma) [8]. Included in Ovalentaria are familiar clades of fishes such as cichlids, blennies, damselfishes, silversides, dottybacks, and mullets. In addition to providing strong support for the monophyly of Ovalentaria, previous phylogenetic studies using DNA sequences sampled from ten nuclear genes discovered that cichlids and the enigmatic Engineer Goby, *Pholidichthys*, are sister lineages. These studies were consistent with traditional taxonomic hypotheses in resolving lineages such as blennies and the atherinomorphs as monophyletic. However, interrelationships among the major lineages of Ovalentaria are not well-resolved, as short branch lengths and poorly supported nodes characterize the earliest divergences in the clade [7, 8, 33, 34]. It is not clear, though, if the lack of resolution among the early diverging lineages of Ovalentaria can be ameliorated through phylogenetic analyses of larger DNA sequence datasets.

Here we employ anchored hybrid enrichment to determine if a phylogenomic dataset provides enhanced resolution of phylogenetic relationships among the major lineages of Ovalentaria, keeping in mind that the AHE kit we used represented the first iteration of the method in this group. After screening for the presence of paralogous loci, which may be have arisen due to the WGD, we inferred phylogenies using over 80,000 bp of DNA sequence data. We also explored the effect of increasing the size of DNA sequence datasets on overall phylogenetic resolution, as measured by average node support across the phylogeny, which included certain key nodes in the Ovalentaria tree.

## Results

**Fig. 1:**
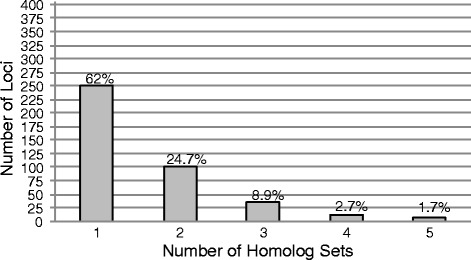
Number of loci captured, and proportion of total captured loci, in each of the five homolog sets

Among the 512 targeted loci 405 were captured for at least four species. There were a total of 638 homolog sets aligned for these 405 loci (see Materials and Methods for details on inference of homolog sets). The number of homolog sets for each locus ranged from 1 to 5, with the number of loci inversely related to the number of homolog sets (Fig. 1). The majority of loci, 62 %, were present in one homolog set, and 86.7 % were present in either one or two homolog sets. The summary statistics on the AHE dataset before manual curation can be found in Table 1. All species had similar statistics, with the exception of *Pholidicthys*, which had fewer contigs and reads in contigs, lower enrichment efficiency, percentage of reads in assemblies, and the number of reads per locus, than the other sampled species.

**Table 1:** Species included in this study, as well as summary statistics from the Anchored Hybrid Enrichment protocol, for each species in the final assembly

Family	Species	Total number of reads	Number of contigs	Reads in contigs	Enrichment efficiency	Number of loci captured	Average locus length	% Reads in assembly	Coverage (reads) per locus	YFTC number	Voucher
**Atherinopsidae**	**Menidia menidia**	9,987,658	763	345,563	273	262	853	3.5	1,319	13569	YPM 20542
**Pseudomugilidae**	**Pseudomugil signifer**	13,797,820	700	295,723	228	260	803	2.1	1,137	21580	YPM 25209
**Aplocheilidae**	**Aplocheilus lineatus**	8,027,160	853	414,328	307	274	788	5.2	1,512	17777	YPM22279
**Fundulidae**	**Lucania goodei**	7,928,592	686	242,710	354	247	771	3.1	983	11543	PW1591
**Belonidae**	**Strongylura marina**	10,242,876	685	282,845	270	274	831	2.8	1,032	23716	Tissued whole
**Chaenopsidae**	**Acanthemblemaria spinosa**	11,755,272	722	374,487	311	270	955	3.2	1,387	12081	PW 1667
**Tripterygiidae**	**Enneanectes altivelis**	10,596,644	837	549,827	464	271	843	5.2	2,029	3249	No Voucher
**Ambassidae**	**Ambassis urotaenia**	13,327,538	1,012	640,383	219	299	984	4.8	2,142	18168	YPM 23178
**Cichlidae**	**Heros appendictulatus**	11,207,474	1,247	404,006	323	257	686	3.6	1,572	19986	ROM 84294
**Cichlidae**	**Retroculus xinguensis**	9,444,720	1,059	315,487	299	223	623	3.3	1,415	11437	PW 227
**Cichlidae**	**Ptychochromis grandidieri**	10,073,544	756	341,896	304	280	878	3.4	1,221	11469	PW 664
**Cichlidae**	**Etroplus maculatus**	12,618,044	797	392,307	278	272	875	3.1	1,442	11521	PW 1333
**Embiotocidae**	**Embiotica jacksoni**	10,992,132	849	600,890	445	293	950	5.5	2,051	17736	PW 2497
**Gobiesocidae**	**Diademichthys lineatus**	11,351,084	968	500,858	431	265	921	4.4	1,890	21699	YPM 25215
**Gobiesocidae**	**Gobiesox maeandricus**	10,827,390	741	462,089	417	250	924	4.3	1,848	15672	SLU-TC 022
**Grammatidae**	**Gramma loreto**	9,002,262	769	384,082	365	275	865	4.3	1,397	21700	YPM 25216
**Mugliidae**	**Mugil cephalus**	11,200,296	902	416,341	263	282	833	3.7	1,476	11546	PW 1602
**Opistognathidae**	**Opistognathus aurifrons**	9,642,318	782	723,920	648	275	867	7.5	2,632	21682	USNM 334483
**Pholidichthidae**	**Pholidichthys leucotaenia**	9,194,340	496	116,744	114	164	604	1.3	712	11559	PW 1659
**Plesiopidae**	**Plesiops coeruleolineatus**	10,783,068	1,122	540,273	449	290	814	5.0	1,863	11481	PW 1012
**Polycentridae**	**Polycentrus schomburgki**	11,935,280	809	440,262	360	280	873	3.7	1,572	12472	PW 1818B
**Pomacentridae**	**Microspathodon bairdii**	12,219,356	899	529,780	300	305	971	4.3	1,737	21686	YPM 25208
**Pomacentridae**	**Pomacentrus nigromanus**	11,243,642	798	421,306	274	290	886	3.7	1,453	12089	PW 1688
**Pseudochromidae**	**Congrogadus subducens**	9,884,860	716	334,900	234	275	846	3.4	1,218	18745	KU 29884
**Pseudochromidae**	**Pseudochromis fridmani**	9,273,084	1,120	564,750	421	292	859	6.1	1,934	23718	ANSP 191950
Bovichtidae	Bovichtus diacanthus	11,205,486	783	493,034	465	286	873	4.4	1,724	3477	No Voucher
Eleginopidae	Eleginops maclovinus	13,566,224	910	605,955	473	291	862	4.5	2,082	7700	YPM 16549
Anomalopidae	Anomalops katoptron	11,624,378	901	591,926	365	318	948	5.1	1,861	13820	YPM 20676
Monocentridae	Monocentrus reidi	15,532,916	923	627,278	289	311	951	4.0	2,017	22123	FMNH 107283

Members of Ovalentaria are highlighted in bold

*YFTC* Yale Fish Tissue Collection number. Voucher codes: *ANSP* Academy of Natural Sciences of Philadelphia, *FMNH* Field Museum of Natural History, *KU* University of Kansas, *PW* Research Collection of Professor Peter Wainwright, UC Davis, *ROM* Royal Ontario Museum, *USNM* National Museum of Natural History

The initial 405 captured loci were reduced to 254 after removing those loci that were missing from more than two of the sampled species. Additional paralogous copies were discovered through inspection of the individual gene trees and distance matrices after the initial filtering of loci using the paralog picker (see Materials and Methods).

**Fig. 2:**
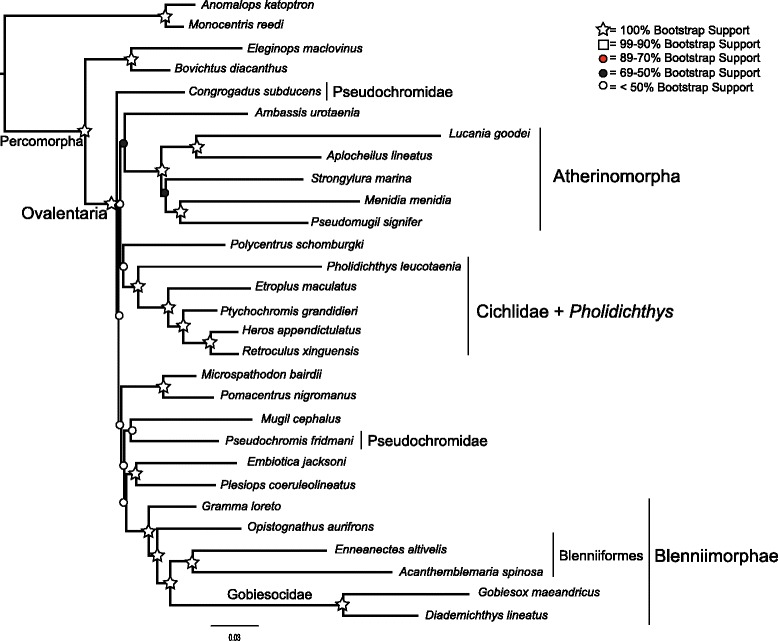
Concatenated maximum likelihood phylogeny inferred using RAxML, from the full 29 species, 107 locus dataset, partitioned by codon position. Shapes and colored circles represent bootstrap support for a given node. Higher-level named clades are noted. Percent GC of third codon positions is listed for each species. Note that Pseudochromidae is not a clade

After removal of all paralogous copies there was 107 loci, totaling 82,782 bp of DNA sequence data (Table 2). In nine cases we used both copies of a particular locus. The full matrix contained 43 % variable sites, and third codon positions comprised 67 % of the variable sites (Table 2). There was a clear bias away from adenine residues at all codon positions. GC%, without accounting for ambiguities is 47.3 %. When accounting for ambiguities, GC% is 52.7 %. G-C skew is−0.051. There was no clear pattern of GC bias in third codon positions (Fig. 2). The compositional homogeneity test implemented in PhyloBayes did not indicate compositional heterogeneity (*p* = 0.11). The principal component analysis (PCA) of the amino acid frequencies did not point to compositional artifacts (not shown). We removed *Pholidicthys* from the PCA because of its large amount of missing data. The full data matrix is available on Dryad (accession pending).

**Table 2:** The number of variable sites in the concatenated dataset, for the whole matrix and for each codon position

	Number of sites	Number of constant sites	Number of variable sites
Whole matrix	82782	47211	35571
1st position	27594	20421	7173
2nd position	27594	23114	4480
3rd position	27594	3676	23918

The length of the individual alignments ranged between 450 and 1605 bp, with a mean of 774 bp (Additional file 1: Table S1). The percentage of missing data for each of the loci, without considering indels, ranged from 1.06 to 25.87 % (Additional file 1: Table S1). When considering only trailing end gaps and missing loci as missing data, the entire concatenated matrix was 90.3 % complete. This decreased to 89.5 % complete when considering indels. The matrix was 96.6 % complete for the number of loci present out of the total 107. The percentage of missing data, average ungapped locus length, ungapped alignment length, and percent presence in the full dataset varied by species (Table 3). The differences in missing data were substantial, ranging from 2.8 % missing for *Anomalops katoptron* to 41 % missing for *Pholidichthys*. Two of the loci that were captured with AHE are frequently used in fish phylogenetics: *Rag1* and *sidkey*. A full list of the loci with their corresponding best BLAST search results can be found in Additional file 2: Table S2.

**Table 3:** The amount of missing data, by species

Family	Species	Percent missing data including trailing ends and indels	Number of loci	Percent presence in matrix	Ungapped alignment length	Ungapped locus length
Anterinopsidae	Menidia menidia	14.4	98	91.6	70,824	723
Pseudomugilidae	Pseudomugil signifer	10.9	104	97.2	73,735	709
Aplocheilidae	Aplocheilus lineatus	8.4	105	98.1	75,857	722
Fundulidae	Lucania goodei	21.4	95	88.8	65,080	685
Belonidae	Strongylura marina	9.0	106	99.1	75,367	711
Chaenopsidae	Acanthemblemaria spinosa	13.0	99	92.5	72,021	727
Tripterygiidae	Enneanectes altivelis	13.1	102	95.3	71,916	705
Ambassidae	Ambassis urotaenia	3.8	107	100.0	79,646	744
Cichlidae	Heros appendictulatus	20.5	102	95.3	65,805	645
Cichlidae	Retroculus xinguensis	28.8	104	97.2	58,933	567
Cichlidae	Ptychochromis grandidieri	6.1	106	99.1	77,747	733
Cichlidae	Etroplus maculatus	8.3	104	97.2	75,914	730
Embiotocidae	Embiotica jacksoni	5.0	105	98.1	78,663	749
Gobiesocidae	Diademichthys lineatus	15.1	94	87.9	74,290	748
Gobiesocidae	Gobiesox maendricus	9.5	100	93.5	74,914	749
Grammatidae	Gramma loreto	3.9	106	99.1	79,574	751
Mugliidae	Mugil cephalus	8.0	105	98.1	76,200	726
Opistognathidae	Opistognathus aurifrons	13.1	102	95.3	71,916	705
Pholidichthidae	Pholidichthys leucotaenia	41.0	97	90.7	48,818	503
Plesiopidae	Plesiops coeruleolineatus	6.9	105	98.1	77,035	734
Polycentridae	Polycentrus schomburgki	4.1	106	99.1	79,382	749
Pomacentridae	Microspathodon bairdii	3.0	106	99.1	80,210	757
Pomacentridae	Pomacentrus nigromanus	3.4	107	100.0	80,008	748
Pseudochromidae	Congrogadus subducens	5.4	105	98.1	78,329	746
Pseudochromidae	Pseudochromis fridmani	5.1	104	97.2	79,601	756
Bovichtidae	Bovichtus diacanthus	3.9	107	100.0	79,562	744
Eleginopidae	Eleginops maclovinus	13.2	100	93.5	71,885	719
Monocentridae	Monocentris reedi	2.9	106	99.1	80,413	759
Anomalopidae	Anomalops kataptron	2.8	107	100.0	80,501	752
	Average	10.5	103	96.5	74,108	717

### Phylogenetic analyses

**Fig. 3:**
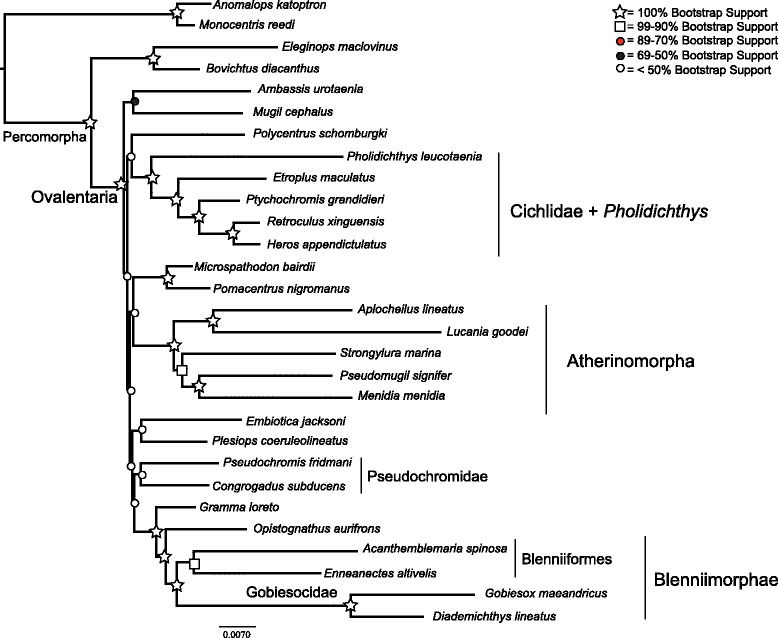
Concatenated maximum likelihood phylogeny inferred using RAxML, from the full 29 species, 107 locus dataset, with 3rd codon positions removed. Shapes and colored circles represent bootstrap support for a given node. Higher-level named clades are noted. Note that Pseudochromidae is a clade, albeit with poor support

**Fig. 4:**
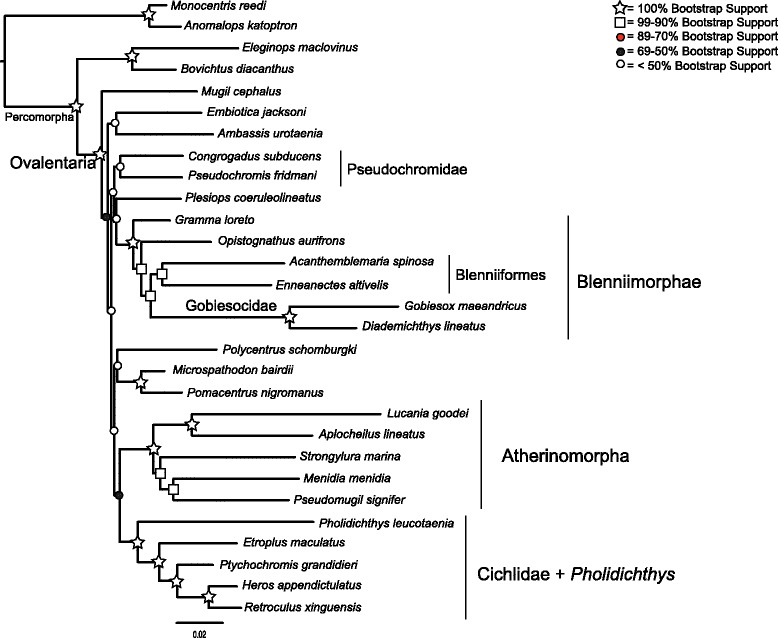
Concatenated maximum likelihood phylogeny inferred using RAxML, from the full 29 species, 107 locus dataset, converted to amino acid sequences, under the JTT substitution model. Shapes and colored circles represent bootstrap support for a given node. Higher-level named clades are noted. Note that Pseudochromidae is a clade, albeit with poor support

**Fig. 5:**
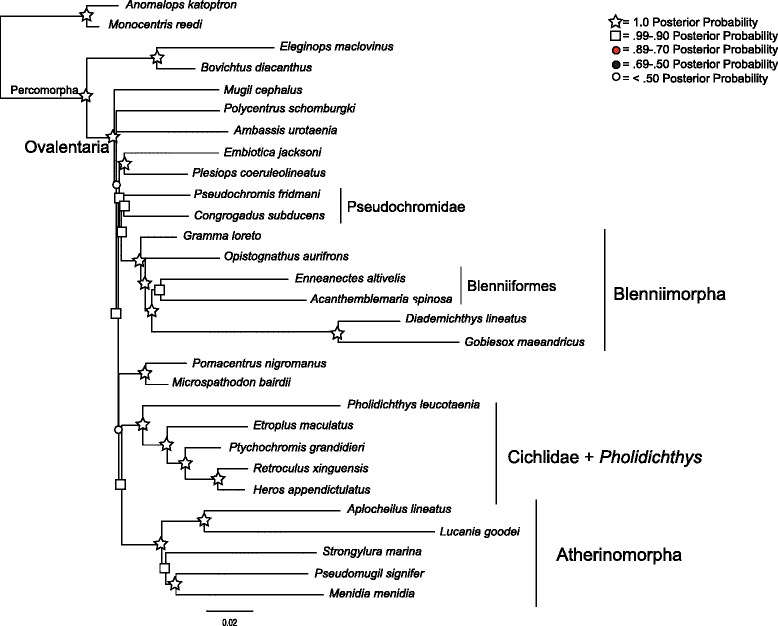
Concatenated phylogeny inferred using the CAT Bayesian mixture model, implemented in PhyloBayes. Shapes and colored circles represent the posterior probability for a given node. Higher-level named clades are noted. Note that Pseudochromidae is a clade, with good support

**Fig. 6:**
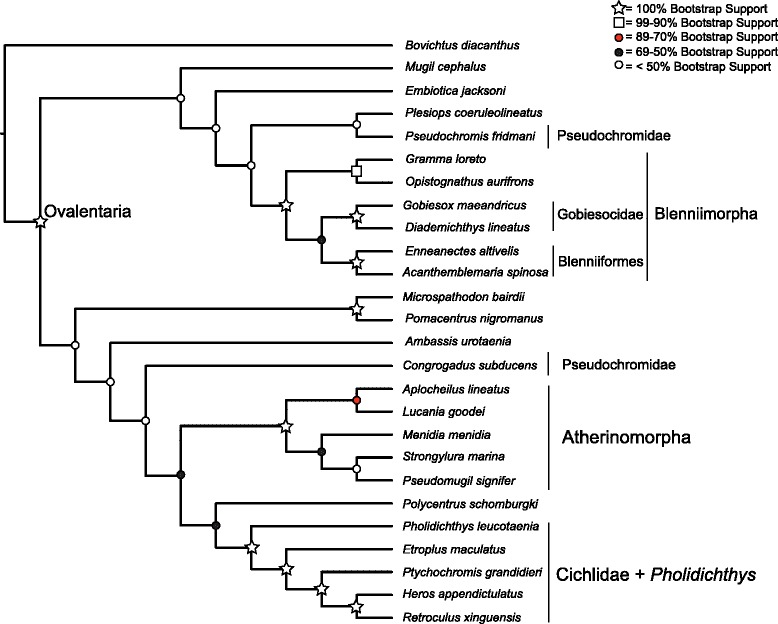
Species tree inferred using MP-EST from the 107 locus dataset. Three outgroup taxa were removed for this analysis, leaving 26 species. Individual gene trees were inferred using RAxML and partitioned by codon position. Shapes and colored circles represent bootstrap support for a given node. Higher-level named clades are noted. Note that Pseudochromidae is not a clade and Atheriniformes is paraphyletic

Partitioning by codon had a much lower AIC score than partitioning by gene (Δ AIC = 38907). The average bootstrap support for the concatenated analyses differed among the partitioning schemes, ranging between 83 % and 76 % (Table 4). Partitioning by gene had the highest average bootstrap support, while phylogenetic analysis of the amino acid translation was lowest. The MP-EST analysis had an average bootstrap support of 69 %. The trees inferred from the full datasets, as well as the species tree, had poor support for the backbone of the phylogeny, with most bootstrap values being less than or close to 50 % (Figs. 2, 3, 4 and 6, Additional file 3: Table S3, Additional file 4: Figure S1, and Additional file 5: Figure S2). However, there were sets of clades that were consistently resolved with high support in all the trees including Ovalentaria, monophyly of cichlids and *Pholidichthys*, the Atherinomorpha, the bleniimorphs, the Blenniiformes, and the Pomacentridae (damselfishes). How these clades relate to one another, or to the other taxa in the analysis was not resolved, as there was very low bootstrap support for nearly all of the other nodes in the tree (Figs. 2, 3, 4 and 6; Additional file 3: Table S3, Additional file 4: Figure S1, and Additional file 5: Figure S2). This included the Pseudochromidae (dottybacks), which did not form a clade when using the full matrix datasets or the species tree analysis, but was resolved as monophyletic in the phylogenies inferred from the dataset with the 3rd codon positions removed and in the tree resulting from analysis of the amino acid matrices, albeit all with poor support (Figs. 3 and 4, and Additional file 5: Figure S2). However, the tree inferred using PhyloBayes provided strong support for this clade (Fig. 5).

**Table 4:** Average bootstrap support and log likelihoods for the different partitioning strategies and analytical methods

Inference strategy	Log likelihood	Average bootstrap support
By Gene	−653571.169	83
By Codon Position	−634117.808	80
3rd Positions Removed	−220968.821	77
Amino Acid Translation	−230313.297	76
MP-EST	n/a	69

The monophyly of Ovalentaria and the clade containing cichlids and *Pholidichthys* were supported with 100 % bootstrap support (Figs. 2, 3, 4, 5 and 6, Additional file 3: Table S3, Additional file 4: Figure S1, and Additional file 5: Figure S2). Relationships within Atherinomorpha and Blenniimorpha varied in support. While atherinomorph monophyly was supported with high bootstrap scores, the interrelationships of the constituent lineages were less well resolved. The Beloniformes (halfbeaks and flying fishes) and Atheriniformes (silversides) were resolved as a clade in all the concatenated analyses (Figs. 2, 3, 4 and 5, Additional file 3: Table S3, Additional file 4: Figure S1, and Additional file 5: Figure S2), but the bootstrap support was <70 % when using the full dataset. However, support for this relationship increased when 3rd codon positions were removed, or when amino acids were analyzed, respectively (supplementary Figs. 3 and 4, Additional file 4: Figure S1). The species tree resolved the Beloniformes nested in Atheriniformes, albeit with low bootstrap support (Fig. 3). In all analyses of the full dataset Beloniformes and Atheriniformes were resolved as a clade that is the sister lineage of Cyprinodontiformes.

The Blenniimorpha and Blenniformes were monophyletic with 100 % bootstrap support in all analyses (Figs. 2, 3 and 5, Additional file 3: Table S3, Additional file 4: Figure S1, and Additional file 5: Figure S2). However, there was poor support for a sister relationship between gobiesocids (clingfishes) and blenniiforms in the species tree analysis (<70 %), but there was stronger support in the phylogenies resulting from analyses of the concatenated dataset. A notable difference between the species tree and phylogenies inferred from the concatenated dataset was the relationship between grammatids and opistognathids (jawfishes). In the phylogenies inferred from the concatenated dataset *Gramma loreto* and *Opistognathus aurifrons* were not monophyletic; however, they were resolved as a clade in the species tree.

The phylogeny inferred from the dataset with reduced taxon sampling resolved the monophyly of cichlids and *Pholidicthys*, monophyly of Atherinomorpha, the sister relationship of Blenniiformes and Gobiesocidae, and monophyly of the Blenniimorpha, with all nodes supported with a 100 % bootstrap value (Fig. 6). The only difference between this reduced dataset and the other matrices was that Cyprinodontiformes and Atheriniformes were resolved as a clade with strong bootstrap support. The phylogeny inferred from the dataset with reduced sampling demonstrates that the number of taxa sampled in the complete matrix was not the driver of poor node support at the base of the Ovalentaria tree. All other relationships in the tree were poorly resolved, as was found with the full datasets.

The phylogeny inferred using PhyloBayes (Fig. 5) provided strong support for the clades listed above, and also resolved a monophyletic Pseudochromidae. It had high support for some of the backbone nodes of the tree, yet it differed topologically from all the trees inferred using maximum likelihood and species tree inferences. The results of the cross-validation test (see Methods) confirmed that the CAT model provided a better fit to the amino acid data than the JTT model (Additional file 6: Figure S3).

### Effect of gene sampling on phylogenetic resolution

**Fig. 7:**
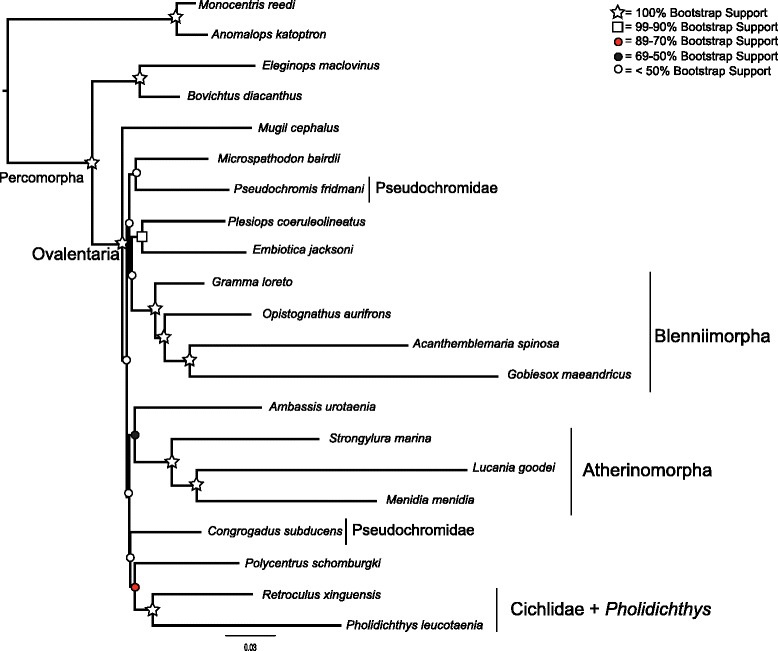
Concatenated maximum likelihood phylogeny, inferred using RAxML from the 107 locus dataset with reduced taxon sampling (21 species), partitioned by codon position. Shapes and colored circles represent bootstrap support for a given node. Higher-level named clades are noted. Note the sister relationship of Cyprinodontiformes and Atheriniformes

**Fig. 8:**
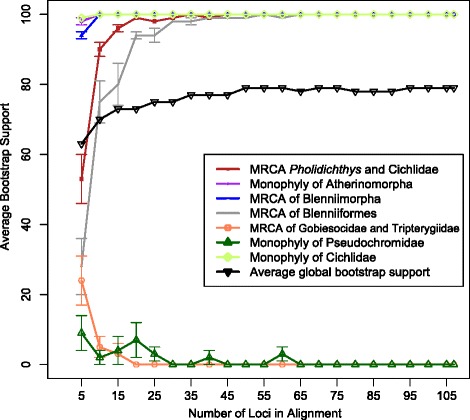
Rarefaction curves displaying the increase in average bootstrap support for maximum likelihood-inferred phylogenies as more data are added to the phylogenetic matrices. The average bootstrap support as data was added to the phylogenetic matrices was also tracked for the following nodes: the monophyly of Cichlidae, the monophyly of Atherinomorpha, the most recent common ancestor (MRCA) of *Pholidichthys* and Cichlidae, the MRCA of chaenopsid blennies and tripterygiid blennies (Blenniiformes), a MRCA of Gobiesocidae and Tripterygiidae, the monophyly of the Pseudochromidae, and the MRCA of Grammatidae, Opistognathidae, Gobiesocidae, and Blenniiformes (Blenniimorpha)

**Fig. 9:**
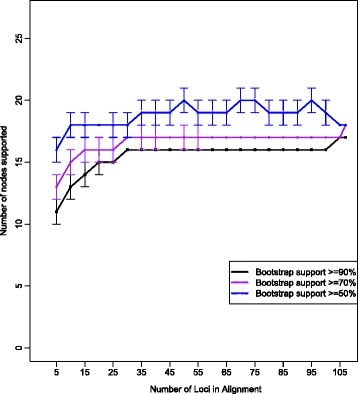
Number of nodes out of the total nodes in the maximum likelihood-inferred phylogenies supported with different bootstrap proportions as more data was added to the analyses

When looking at the rarefaction curves, the average global bootstrap support value started at 64 % and slowly increased as more loci were added, plateauing at 78–80 % once 35 loci were included (Fig. 7). Bootstrap support for monophyly of cichlids, atherinomorphs, and Blenniimorpha was over 90 % with the inclusion of five loci and reached 100 % when ten loci were sampled. Bootstrap support for the clade containing *Pholidicthys* and cichlids and monophyly of Blenniiformes increased as loci were added and both nodes were supported with 100 % bootstrap scores once 55 loci were included (Fig. 7). Support for a monophyletic Pseudochromidae was low when few loci were included and decreased to zero once 30 loci were added. Similarly, support for a clade containing gobiesocids and tripterygiids (triplefin blennies) quickly went to zero as more loci were included. The rarefaction curve that tracked number of nodes with bootstrap values appeared to reach a plateau, although the number of nodes with greater than 50 % bootstrap support was not stable (Fig. 8). The number of nodes with greater than 70 % and 90 % bootstrap support plateaued with the inclusion of 30 loci, although there was a slight uptick in the 70 % and 90 % nodes when all 107 loci were included (Fig. 9).

## Discussion

The promise of phylogenomics is that the ability to collect many orthologous loci for taxa of interest will increase the resolution of the Tree of Life, even for nodes that have historically been difficult to resolve with certainty. Consistent with other studies [35] we show that this is not necessarily the case for all clades. Results from our analyses provide independent confirmation of Ovalentaria monophyly, and also strongly support the phylogeny that used a much smaller set of loci, including the resolution of the enigmatic engineer gobies (*Pholidichthys*) as the sister lineage of cichlids, thus proving the robustness of the loci captured using AHE. However, even with a large amount of sequence data, and after accounting for paralogous gene copies, both maximum likelihood and species tree analyses failed to resolve many nodes in the Ovalentaria phylogeny, while the PhyloBayes analysis of the amino acid matrix was more successful. Our results support the growing recognition that the application of phylogenetic models to phylogenomic scale datasets may not always capture the increased complexity that underlies the data [31, 36, 37]. For example, we found that at a certain point increasing the number of loci in the dataset did not result in increased average node support. Our results underscore that the advent of phylogenomics must also be accompanied by methods to better analyze these complex datasets [38], as some systematic errors in phylogenomic datasets may be difficult to avoid.

### Ovalentaria Interrelationships

An advantage of inferring phylogenies from multiple loci is that the repeatability of clades among individual gene trees provides confidence in the phylogenetic resolution [39]. Our results confirmed the results of previous studies that support monophyly of Ovalentaria that was intimated in phylogenetic analyses using mitochondrial or nuclear genes [34, 39–42]. Our results also provide an independent corroboration of the monophyly of *Pholidichthys* and cichlids [7, 8, 33], which was resolved with 100 % bootstrap support after using just a small portion of the total number of markers (Fig. 7). The inclusion of the cichlids as a positive control was successful, as the cichlid interrelationships agreed with previous phylogenetic hypotheses [43, 44], although no African cichlid species were sampled in the AHE dataset. Notably, none of the trees we inferred supported a “chromide” clade of damselfishes, surfperches, and cichlids. The existence of a chromide clade has been a longstanding phylogenetic hypothesis [33, 45], but we find no evidence for it here. However, the PhyloBayes tree did recover a monophyletic pseudochromid clade. However, of all recent large-scale analyses of percomorphs, this was the only one to do so [7, 8, 33]. Although outside the scope of this study, investigating the cause of this incongruence between our and other studies represents an important step towards resolving this node in the percomorph tree of life.

Phylogenetic analyses of the AHE dataset resolved Atherinomorpha as a clade, confirming both morphological and molecular studies that have long recognized their monophyly [8, 33, 40, 46–50]. However, different analyses resulted in different relationships among the Beloniformes, Atheriniformes, and Cyprinodontiformes. The phylogenies inferred using maximum likelihood resolved Atheriniformes and Beloniformes as a clade (Figs. 2, 3 and 4, Additional file 3: Table S3, Additional file 4: Figure S1, and Additional file 5: Figure S2), while the analysis with reduced taxon sampling resolved Cyprinodontiformes and Atheriniformes as a clade with 100 % bootstrap support (Fig. 6). The phylogenetic discordance observed among these analyses, as well as between ours and those of the previous morphological and molecular studies [8, 33, 40, 46–50] may be due to gene tree heterogeneity and deep coalescence among the major lineages of Atherinomorpha.

The Blenniimorpha has only recently been delimited through the use of molecular data [7, 8, 33]. Our study is an independent corroboration of this result. Some of the interrelationships in the Blenniimorpha were initially hypothesized from analyses of morphological and molecular data [42, 51–53]. The monophyly of the Blenniiformes has been called into question [7], but we find that they form a clade sister to gobiesocids, in agreement with other molecular and morphological studies [8, 33, 39, 51, 54].

### The anchored hybrid enrichment dataset

The anchored hybrid enrichment method provided DNA sequences from hundreds of loci. However, for any individual species there were ca. 50 % as many markers than the 512 loci from Lemmon et al. (2012). This result, however, is not unexpected. The Lemmon et al. (2012) study found a similar result when sequencing the killifish, *Heterandria formosa*, which is a member of Ovalentaria and shares common ancestry exceeding 250 Ma with the model organism used to make the probe set, *Danio rerio* [6]. Thus, our capture results are within the expected range, given the divergence time between our clade of interest and the model teleost used to design the kit. Successful enrichment depends on the divergence time to the model species [22, 55]. The best way to deal with low capture rates is to design probe sets using taxa that are closer phylogenetically to the clade being investigated [22]. These new capture kits have already successfully completed numerous fish data sets. Some species, such as *Pholidicthys* tended to have short sequence lengths. In these cases the only area of the locus sequenced was primarily the anchor region in the center. This is because for species with poor capture efficiency, the coverage will be lower. Since the coverage is highest in the probe region and lower in the flanks, low coverage will cause the flanks to drop out first (e.g., primarily probe regions will remain). New probe designs that use multiple neighboring anchor regions should ameliorate this. The benefit of collecting data using methods such as AHE is that there will be enough loci captured that those that are poorly sequenced, or uninformative, can be eliminated from the data matrix [22, 24].

### The Performance of the AHE dataset

We collected over 80 kb of DNA sequence data in our study. Nonetheless, there was a lack of phylogenetic resolution among the major lineages of Ovalentaria. Other studies have shown that adding more sequence data increased node support, even in deep and rapid radiations [17, 19, 20, 36, 56, 57]. In a similar situation to ours, and with a similar number of taxa, McCormack et al. [9] used a phylogenomic dataset to resolve interfamilial relationships of Neoaves. They found that increasing their dataset from 416 to 1541 loci significantly improved average maximum likelihood bootstrap support, which was already high. However, there were still a substantial number of poorly resolved nodes in their 1541 locus maximum likelihood tree (Additional file 3: Table S3).

Wagner et al. [16] showed a striking example of the power of large datasets. Using millions of base pairs of DNA sequence data, collected using RAD-seq, they resolved the interrelationships among very recently diverged cichlid species. However, in their case they needed ~300,000 bp of data before individual species were reciprocally monophyletic. Support and resolution increased nearly linearly until ~2,000,000 bp, when it began to plateau. This result suggests that it may take a very large amount of data to solve difficult phylogenetic problems, far more than we have employed here. However, Wagner et al. (2013) were contending with very shallow genetic divergences and rampant incomplete lineage sorting between species. They were using a very large proportion of these fishes’ genomes to extract a signal of differentiation. In contrast, Ovalentaria contains several well-differentiated lineages, but their interrelationships are poorly known because there is little phylogenetic resolution among a set of short internodes that may reflect a history of rapid lineage diversification. However, it is also possible that these short internodes are the result of shifts in rates of molecular evolution across the tree [58].

In Ovalentaria, average support and number of nodes supported plateaus at a relatively low value before the majority of available loci are sampled (Figs. 7 and 8). This pattern was also observed by Rodríguez-Ezpeleta [59], which they attributed to systematic error leading to non-phylogenetic signal. This may occur when sequences are saturated, causing a large number of homoplasious nucleotide substitutions, or when there is model misspecification [31]. One of the pitfalls of phylogenomics is the potential for the inference of a strongly supported, but erroneous, phylogeny because systematic error increases as more data is used [24, 35]. Such error does not appear to be confounding the support for monophyly of major Ovalentaria lineages, as many of these clades are consistently supported in several other phylogenetic studies [8, 33, 34, 40, 42, 49, 54]. However, stochastic error, systematic error and non-phylogenetic signal can also lead to low node support [12, 59], as observed in the lack of phylogenetic resolution among the major lineages of Ovalentaria.

In the case of “stochastic error” it may have been that the probe set used for Anchored Hybrid Enrichment most likely captured loci sharing similar attributes. The similarity of these loci to one another could cause the lack of resolution we have in our dataset, if they are all biased. However, we find this to be unlikely, as other studies using this method do not appear to show a clear pattern of bias [60]. We qualify this by writing that without sampling other classes of markers throughout the genome, it is difficult to say for certain. However, our rarefaction curves suggest that for certain approaches to phylogenetic inference such as the likelihood analyses used here, simply adding more loci, at least those captured using this first version of the Anchored Hybrid Enrichment method, will not increase support after a certain number of loci are added.

### Systematic error?

Increased taxon sampling has been shown to increase the accuracy and node support of phylogenetic trees [31, 61–65]. In our study we tested the inverse of this: to determine if removal of taxa would lead to decreased support at nodes (see Methods). With the exception of the interrelationships of the Atherinomorpha, it mostly did not change node support. This is most apparent with the monophyly of *Pholidichthys* and cichlids, an unexpected relationship when first discovered, and one which may have been due to the relatively large number of cichlids in that study (Wainwright et al. [33]). Thus, the inclusion of multiple cichlid species may be expected to account for the high support of that node. Removing all but one cichlid had no effect on node support, indicating that adding additional taxa for each of the Ovalentaria lineages would not necessarily increase support for nodes at the base of the tree. This suggests that adding more taxa would simply add more well-supported apical nodes to each of the major Ovalentaria lineages, assuming we have sampled all basal Ovalentaria lineages [19].

Another strategy to minimize non-phylogenetic signal is to remove fast evolving sites, which will reduce the number of characters affected by multiple substitutions [12, 31, 36, 59, 66]. This is done by either substituting slow-evolving for fast-evolving taxa, or removing third codon positions [12, 67]. The problem of substantial molecular evolutionary rate heterogeneity does not appear to be present in the Ovalentaria AHE dataset. In fact, the lineages and species with the longest reconstructed branch lengths were resolved in well-supported clades that agree with previous studies using different datasets, such as in the Blenniimorpha. In addition, there was essentially no change in the inferred phylogeny when 3rd codon positions were removed, but there was a decrease in average bootstrap support (Additional file 4: Figure S1).

The remaining sources of systematic error are incorrect identification of orthologs and model misspecification. The paralog picker was not perfect; there were still alignments with apparent paralogous gene copies after its application. Detection of non-orthologous loci is difficult, but can be automated to a certain degree, like we have here [68, 69]. However, manual inspection of each alignment and individual gene tree was still necessary to filter out all the paralogous gene copies. This was time consuming and will prove cumbersome as phylogenomic datasets continue to increase in size.

Model misspecification is more difficult to address. It is not clear which models are best for large, complex datasets, especially those of coding sequence, or how to partition these datasets. Our dataset was too large to use in PartitionFinder [70], so we opted for several obvious partitioning schemes and used the most complex substitution model for each partition, as topological inference has been shown to be robust to model over-parameterization [71]. However, with phylogenomic data, the question of model adequacy becomes increasingly relevant. More sophisticated substitution models such as the site-heterogeneous CAT model [72] have been shown to deal well with non-phylogenetic signal [12], and it did provide better results for our data than the other models. It is not clear, though, if this was due to a large proportion of non-phylogenetic signal in our data, as there was no obvious trend towards this. Our results highlight the need for theoretical studies developing new approaches for data modeling and investigations into the influence of model misspecification in genomic scale datasets [38].

An alternative source of conflict is that individual gene histories can deviate from the true species tree, especially when successive speciation events have been rapid, including those at scales of deep evolutionary time [73]. The molecular phylogenies of Ovalentaria exhibit a signal of rapid diversification among the major constituent lineages, that is, short internodes at the base of the tree coupled with long terminal branches [15]. This leads to the expectation of heterogeneity among the individual gene trees [15]. The poor resolution at the base of each of the Ovalentaria gene trees appears to be due to a lack of signal in any one particular locus. As such, it appears that weak support at the base of the Ovalentaria species tree is not due to significant discordance among individual gene tree histories. However, the gene trees would be discordant with little signal if they just reflected a great deal of uncertainty. That said, that discordance would not be reflected by a pattern of strong support for alternate topologies. The method we used to infer a species tree, MP-EST, takes as input individual gene trees. This results in a species tree that is only as robust as the gene trees provided. Nonetheless, there was clearly phylogenetic signal for several major clades in each of the individual gene trees, as much of the well-supported parts of the species tree topology agreed with the concatenated datasets (Figs. 2, 3, 4 and 5, Additional file 3: Table S3, Additional file 4: Figure S1, and Additional file 5: Figure S2).

Although there are cases where the low support in individual gene trees can be ameliorated by the concatenation of all loci [74] this was not the case in our dataset. While the difference in bootstrap support values between the species tree and those of the concatenated ones suggested that the simple addition of data did help to promote some increased resolution, the increased bootstrap support values did not lead to high values for previously poorly-supported clades. We believe the reason to be that we simply have a very difficult phylogenetic problem that will be difficult to solve, as our rarefaction curves suggest. Although future probe sets that capture longer loci that may increase support for individual gene trees, and perhaps the entire concatenated matrix, the increased resolution of the PhyloBayes based topology suggests that better models, and not more data are critical towards our ability to successfully resolve a Genomic Tree of Life.

## Conclusions

The phylogenetic analyses of more than 100 loci to infer the relationships of the acanthomorph teleost clade Ovalentaria demonstrates that some, but not all, lineages connected by short internodes may avoid resolution under certain analytical conditions. The lack of resolution among the major lineages of Ovalentaria did not appear to result from a shortage of loci, as demonstrated by the rarefaction curves. After accounting for paralogous gene copies and attempting to minimize missing data, we had substantially fewer loci than the 512 that were targeted. This was not unexpected given the long divergence time between Ovalentaria and the model teleost used in this first version of the AHE capture kit, as well as the teleost-specific whole genome duplication event. The AHE dataset provided robust phylogenetic inference, as it validated the results of previous phylogenies that used different sets of markers. Our results highlight the need for new models to accommodate increasingly large and more complex phylogenomic datasets, as only one analytical method was able to provide resolution of relationships across the Ovalentaria tree. We hope that improved models, as well as new kit designs and bioinformatic strategies for phylogenomic data collection and analysis, will ultimately facilitate estimation of well-resolved phylogenies of all clades in the Tree of Life.

## Methods

### Taxon sampling

The phylogenies from Wainwright et al. [33] and Near et al. [8] were used to choose species for this study. Taxa were chosen so that they sampled all major lineages in Ovalentaria, making sure to capture nodes deep in the clade. In addition, several nodes with closely related species were sequenced (Table 1). These served as positive controls to help detect paralogous gene copies, as well as to assess the effect of taxon sampling on node support (see below). If we did have paralogs we might expect, for instance, that the two damselfish species would fall out on opposite ends of the tree with high support.

### DNA extraction, library preparation, sequencing, read assembly, assessment of paralogous loci, and pair-wise sequence alignment

DNA was extracted from fish tissues preserved in 70–95 % ethanol or were obtained from museum collections. Genomic DNA was extracted from muscle or fin clips using a DNeasy Tissue Extraction Kit (Qiagen, Valencia, CA). Total amounts of DNA were measured using a NanoDrop (Thermo Scientific). Data were collected at the Center for Anchored Phylogenomics at Florida State University (www.anchoredphylogeny.com). Library preparation, enrichment, sequencing, and the probe set used followed the protocols of Lemmon et al. [22].

The reads from the sequencing run went through three processing steps before they were used for phylogenetic analyses using in house programs written in Java (Dryad Accession #): first an assembly step, then use of an automated algorithm to filter out paralogous loci, followed by assembly and manual curation of contigs. The assembly was performed on all of the loci simultaneously, with one seed/alignment per locus. First, a set of reads for a given individual was mapped to a reference using spaced Kmers that allow for 45 % sequence divergence. The best-matching read was chosen, and the other reads were aligned to this best-matching read, with a requirement of 95 % similarity in the overlapping region, which had a minimum overlap of 20 bp. It is important to note that reads coming from paralogous gene copies were not typically aligned at this step. A majority-rule consensus sequence was taken from this alignment, with the minimal requirement of 2x coverage. This consensus sequence was used as the seed in the next step. An extension assembly was conducted in which each seed was “grown” outward, using the reads that overlap with 95 % agreement with the seed from the previous step. Another consensus sequence was taken once the alignment could no longer be “grown” outward, using reads that overlapped with 95 % agreement with the seed. This was done using multiple passes through the read file, if necessary. The raw read file was reduced in the last step by removing the reads already present in the alignment. The process outlined here was repeated N number of times, to produce N consensus sequences, each representing different putatively orthologous genes. A locus was considered “captured” if a consensus sequence length of >350 bp was recovered in any of the assembly rounds.

After the assembly step, orthologous genes were identified using a “paralog picker” algorithm. The “paralog picker” is preferred because many other methods such as OrthoMCL assume that the sequences are protein coding [75]. Not all of the anchor loci are, so these methods will not allow us to apply a consistent method across all of our loci.

The steps of the paralog picker are as follows: First, a reference individual was chosen, which was typically the one that exhibited the best capture efficiency. Second, the consensus sequences from each individual were aligned to the reference individual’s sequences. Third, we defined the first homolog set as the first reference sequence, which is the first homolog identified for individual 1 for the locus, and the sequences from each individual that best aligned to the reference sequence. Fourth, the sequences assigned to the first homolog set were removed. Fifth, a second homolog set was defined as the second reference, which may be the second copy of a duplicated gene, and the sequences from each individual that best aligned to that reference sequence (after excluding those sequences that were chosen for the first homolog set). Sixth, sequences assigned to the second homolog set were removed. This six-step procedure was repeated until all homolog sets were used up.

After execution of the “paralog picker,” multiple sequence alignments were conducted for each ortholog set using MUSCLE [76], implemented in Geneious v5.6.4, created by Biomatters. Available from http://www.geneious.com/. Alignments with greater than two missing species were discarded and then trimmed at the 5′ and 3′ ends to reduce the amount of missing data. If greater than a third of the taxa in the alignment had missing data, then the data was trimmed until less than half of the species had missing data.

Alignments were discarded unless they were at least 450 base pairs long, with 150 base pairs present for the species in the alignment with the shortest sequence. Alignments were manually curated to put all the sequences into open reading frames. For each alignment, pairwise distances among sequences were calculated to identify sequences that exhibited unusually high divergences, which were removed.

Phylogenies were inferred from each locus using MrBayes 3.2 [77]. Two runs for each gene with four chains each were performed, each for 10,000,000 generations. Default priors on cladogenesis were used and the GTR + Γ model of nucleotide substitution was used for all runs, partitioning by codon position. We decided on the GTR + Γ model by running PartitionFinder on a subset of loci, but not all, to reduce computational burden. The GTR + Γ model was inferred for these sequences. Convergence of the model parameters sampled by the chains was assessed using Tracer v1.5 and convergence of topologies was assessed using the “cumulative” and “compare” functions in AWTY [78]. Individual gene trees were inspected for the presence of paralogous gene copies, primarily through unusual and strongly supported phylogenetic resolutions or if a taxon exhibited a very long branch in the gene tree compared to other species. In all cases where there were putative paralogous gene copies, the “offending” sequences were removed from the alignment, and the locus was discarded if the editing resulted in more than two missing species. The reference sequence for each locus was then compared to sequences in GenBank using BLAST searches [79].

### Phylogenetic analyses

Phylogenies were inferred from each locus using MrBayes 3.2 [77]. Two runs for each gene with four chains each were performed, each for 10,000,000 generations. Default priors on cladogenesis were used and the GTR + Γ model of nucleotide substitution was used for all runs, partitioning by codon position. We decided on the GTR + Γ model by running PartitionFinder on a subset of loci, but not all, to reduce computational burden. The GTR + Γ model was inferred for these sequences. Convergence of the model parameters sampled by the chains was assessed using Tracer v1.5 and convergence of topologies was assessed using the “cumulative” and “compare” functions in AWTY [78]. Individual gene trees were inspected for the presence of paralogous gene copies, primarily through unusual and strongly supported phylogenetic resolutions or if a taxon exhibited a very long branch in the gene tree compared to other species. In all cases where there were putative paralogous gene copies, the “offending” sequences were removed from the alignment, and the locus was discarded if the editing resulted in more than two missing species. The reference sequence for each locus was then compared to sequences in GenBank using BLAST searches [79].

Phylogenetic trees were inferred using two methods: maximum likelihood analyses of a dataset where all genes were concatenated and a species tree reconstruction. Four different partition schemes were applied to the sets of concatenated alignments; partitioned by gene, partitioned by codon position, portioned by gene with all third codon positions removed, an alignment consisting of only 3rd codon positions, and two different alignments of the translated amino acid sequences. The AIC was used to decide between partitioning by gene vs. by codon position for further analyses (see below). We also analyzed the full amino acid matrix using a Bayesian mixed model analysis, implemented using the CAT model in PhyloBayes. We summarized the PhyloBayes trees when the “maxdiff” between the chains was <0.1. We further analyzed the amino acid matrix in PhyloBayes through the use of a compositional homogeneity test [80]. We also performed a principle component analysis of the amino acid frequencies to test for compositional artifacts. We removed *Pholidicthys* from the PCA because of its large amount of missing data.

We performed a statistical model comparison using the cross-validation (CV) method available in PhyloBayes to statistically test that the CAT model was a better fit to the data than the JTT model. A learning and a test set were generated by randomly splitting the original alignment into 10 replicates made of 90 % and 10 % of the original sites, respectively. Each 90 % dataset was run with pb_mpi for 50,000 generations subsampling every 10, with a burnin of 5000 and the specified model. The 10 % datasets were then used to cross-validate each run using readpb_mpi’s cv option.

We tested the effect of taxon sampling on node support by removing closely related species from the dataset(Additional file 7: Table S4). The new dataset contained one cichlid, one gobiesocid, one bleniiform, one pomacentrid, and one of each of the three atherinomorph lineages, for a total of 21 species in the subsampled dataset, which was partitioned by codon position. The phylogenies for the concatenated nucleotide sequence datasets were inferred by using RAxML 7.2.6 with the default GTR+ Γ model for each of the nucleotide data partitions and JTT + Γ model for the amino acid alignment, the latter chosen using ProtTest v3.4 [81, 82]. We also inferred a tree using the Dayhoff model of AA substitution, as it is more sensitive to compositional bias. Support for nodes in the RAxML inferred trees was assessed using a rapid bootstrap analysis (option -f a) with 500 replicates. Note that the version of RAxML we used in our analyses no longer overrides the gamma model when doing a rapid bootstrap, and does not default to the CAT approximation.

We tested the effect of step-wise addition of more data on average node support using rarefaction curves constructed for the concatenated datasets. These rarefaction curves were made by randomly sampling from the total pool of loci in our dataset. We partitioned by codon position because this allowed us to make use of all the data while minimizing the number of parameters requiring estimation. The random sampling was done in increments of five loci to make increasingly longer concatenated datasets. The random sampling was performed twenty times for each sampled pool of loci and average bootstrap support for each tree was calculated for each sampling pool. We also calculated average bootstrap support for certain key nodes in each of the datasets including the most recent common ancestor (MRCA) of *Pholidichthys* and Cichlidae [7, 8, 33], the MRCA of chaenopsid blennies and tripterygiid blennies [8, 33, 54], a MRCA of gobiesocids and tripterygiids (Betancur-R. et al. 2013), the monophyly of the Pseudochromidae because previous results have not resolved them as a clade [8, 33], and the MRCA of the clade containing Grammatidae, Opistognathidae, Gobiesocidae and Blenniformes [6, 7, 33]. As “positive controls” the monophyly of both Cichlidae and Atherinomorpha were tracked. In this case, “positive controls” were groups of fishes that have been accepted to be clades. This was done to ensure that the results of the rarefaction curve were robust.

In addition to average node support, rarefaction curves were constructed for average number of nodes with bootstrap values greater than 50, 70, and 90 % bootstrap support. Code for constructing the randomized datasets, as well as for distributing large numbers of RAxML jobs across numerous computer nodes is available on Dryad (accession pending).

Species trees were inferred using MP-EST [83]. MP-EST takes as input maximum likelihood estimated gene trees. Partitioned maximum likelihood analyses using RAxML were used to infer each gene tree. MP-EST requires rooted trees and can only use one outgroup. We used *Bovichtus diacanthus* as the rooted outgroup, removing the three other outgroup species. A species tree was inferred using MP-EST from the bootstrapped gene trees, which incorporates phylogenetic uncertainty into the analysis. The resulting set of species trees were summarized using SumTree, implemented in Dendro-Py [84].

## Additional files

Additional file 1: Table S1. Percentage of missing data, by locus. Available Online. Additional file 2: Table S2. Best BLAST hits for the 107 locus dataset. Available Online. Additional file 3: Table S3. Concatenated maximum likelihood phylogeny inferred using RAxML, from the full 29 species, 107 locus dataset, partitioned by gene. Shapes and colored circles represent bootstrap support for a given node. Higher-level named clades are noted. Note that Pseudochromidae is not a clade. Additional file 4: Figure S1. Concatenated maximum likelihood phylogeny inferred using RAxML, from the full 29 species, 107 locus dataset, inferred using only 3rd codon positions. Shapes and colored circles represent bootstrap support for a given node. Higher-level named clades are noted. Note that Pseudochromidae is not a clade. Additional file 5: Figure S2. Concatenated maximum likelihood phylogeny inferred using RAxML, from the full 29 species, 107 locus dataset, converted to amino acid sequences, under the Dayhoff substitution model. Shapes and colored circles represent bootstrap support for a given node. Higher-level named clades are noted. Note that Pseudochromidae is a clade, albeit with poor support. Additional file 6: Figure S3. Results of cross-validation test. Additional file 7: Table S4. Species removed for reduced taxon sampling analysis. Available online.
