# Supplementary material for: Are 100 enough? Inferring acanthomorph teleost phylogeny using Anchored Hybrid Enrichment
Source: BMC Evol Biol. 2015 Jun 14;15:113. doi: 10.1186/s12862-015-0415-0 (PMC4465735; doi:10.1186/s12862-015-0415-0)
Supplement: Additional file 1: Table S1. — Percentage of missing data, by locus. Available Online. [file 12862_2015_415_MOESM1_ESM.pdf]

Locus	Sequence Length	Number of Sequences	Percent Missing (Trailing Ends)
10.1	528	27	8.1
15.1	741	27	11.7
24.1	798	28	7.0
27.1	507	29	16.2
34.1	819	28	12.0
37.1	765	27	16.4
38.1	693	28	8.5
44.1	627	29	7.6
49.1	510	28	7.3
58.1	606	27	8.9
67.1	600	27	10.7
69.1	564	28	7.8
73.1	651	29	7.8
78.1	744	29	4.8
81.1	834	28	12.7
81.2	669	29	12.2
91.2	762	29	7.4
94.1	645	27	16.7
95.1	735	28	9.0
97.1	720	29	1.6
98.1	789	27	11.0
99.1	450	28	6.8
101.1	480	28	7.5
103.1	516	27	10.6
109.1	639	28	7.7
113.1	612	28	8.5
117.1	861	28	6.6
117.2	783	27	12.6
120.1	684	28	4.2
126.1	711	28	21.5
129.1	846	28	6.4
129.2	588	27	14.2
135.1	708	28	5.5
136.1	1,086	29	4.7
137.1	1,029	27	14.1
138.1	720	29	3.5
143.1	627	29	1.1
148.1	465	28	3.7
152.1	510	29	1.8
157.1	699	27	9.9
160.1	1,479	28	8.2

161.1	1,515	27	10.2	
166.1	1,026	27	17.9	
173.1	1,140	27	10.1	
181.1	546	28	4.8	
188.1	840	28	9.0	
189.1	873	29	7.5	
190.2	729	28	7.5	
193.1	771	28	14.7	
195.1	729	29	6.2	
202.1	792	29	4.9	
211.1	705	28	10.3	
211.2	852	27	12.7	
221.1	849	28	25.9	
230.1	885	28	9.1	
231.1	1,005	28	4.9	
232.1	801	29	4.0	
235.1	744	28	9.8	
235.2	741	29	1.7	
240.1	1,176	29	19.4	
245.1	618	27	12.3	
250.1	888	28	7.8	
257.1	462	27	16.1	
261.1	555	29	5.2	
268.1	618	27	20.4	
269.1	657	27	8.9	
269.2	723	29	2.8	
271.1	843	29	6.2	
292.1	609	29	2.0	
311.1	531	29	13.9	
314.1	741	27	14.3	
316.1	474	29	4.1	
325.1	867	28	8.8	
325.2	888	28	10.3	
328.2	477	27	11.0	
328.3	480	28	13.1	
329.1	489	29	5.4	
336.1	993	27	17.4	
344.1	822	29	9.8	
351.1	1,149	28	9.8	
362.1	630	28	9.6	
375.1	1,350	29	9.9	
376.1	1,605	29	18.0	

382.1	675	29	5.1
386.1	789	27	14.6
390.2	471	28	19.0
400.1	786	28	11.3
418.1	714	29	2.4
424.3	627	27	10.8
435.1	600	27	18.8
446.1	894	29	2.1
447.1	702	29	1.5
454.1	1,404	28	7.1
455.1	1,401	27	14.0
469.1	627	28	3.8
469.4	663	29	13.6
474.1	1,515	29	10.4
479.1	900	28	7.3
480.1	1,566	27	16.1
481.1	732	28	5.8
484.1	708	28	10.1
488.1	798	28	13.0
492.1	492	29	1.8
496.1	525	27	9.9
501.3	750	27	15.6
503.1	711	27	8.5
512.1	744	29	2.2
**Mean**	**774**	**28**	**9.7**

I28_Monocentridae_Monocentrus_reidi_22123

525

I29_Anomalopidae_Anomalops_katoptron_13820

525

I3_Chaenopsidae_Acanthemblemaria_spinosa_23716

426

I4_Fundulidae_Lucania_goodei_11469	I5_Aplocheilidae__Aplocheilus_lineatus_21580
525	525

I6_Belonidae_Strongylura_marina_11543

I7_Mugliidae_Mugil_cephalus_15672

I8_Embiotocidae_Embiotica_jacksoni_11437

I9_Opistognathidae_Opistognathus_aurifrons_21700

Species
I1_Atherinopsidae_Menidia_menidia_17777
I10_Polycentridae_Polycentrus_schomburgki_11559
I11_Grammatidae_Gramma_loreto_21699
I12_Ambassidae_Ambassis_urotaenia_13569
I13_Gobiesocidae_Gobiesox_maeandricus_17736
I14_Gobioscocidae_Diademichthys_lineatus_11521
I15_Pomacentridae_Microspathodon_bairdii_11481
I16_Pomacentridae_Pomacentrus_nigromanus_12472
I17_Pseudochromidae_Congrogadus_subducens_21686
I18_Pseudochromidae_Pseudochromis_fridmani_12089
I19_Plesiopidae_Plesiops_coeruleolineatus_21682
I2_Pseudomugilidae_Pseudomugil_signifer_18745
I20_Cichlidae_Heros_appendictulatus_3249
I21_Cichlidae_Retroculus_xinguensis_19986
I22_Cichlidae_Ptychochromis_grandidieri_18168
I23_Cichlidae_Etroplus_maculatus_12081
I24_Pholidichthidae_Pholidichthys_leucotaenia_11546
I25_Tripterygiidae_Enneanectes_altivelis_23718
I26_Bovichtidae_Bovichtus_diacanthus_3477
I27_Eleginopidae_Eleginops_maclovinus_7700
I28_Monocentridae_Monocentrus_reidi_22123
I29_Anomalopidae_Anomalops_katoptron_13820
I3_Chaenopsidae_Acanthemblemaria_spinosa_23716
I4_Fundulidae_Lucania_goodei_11469
I5_Aplocheilidae__Aplocheilus_lineatus_21580
I6_Belonidae_Strongylura_marina_11543
I7_Mugliidae_Mugil_cephalus_15672
I8_Embiotocidae_Embiotica_jacksoni_11437
I9_Opistognathidae_Opistognathus_aurifrons_21700
