# Supplementary figures and images for: Are 100 enough? Inferring acanthomorph teleost phylogeny using Anchored Hybrid Enrichment
Source: BMC Evol Biol. 2015 Jun 14;15:113. doi: 10.1186/s12862-015-0415-0 (PMC4465735; doi:10.1186/s12862-015-0415-0)

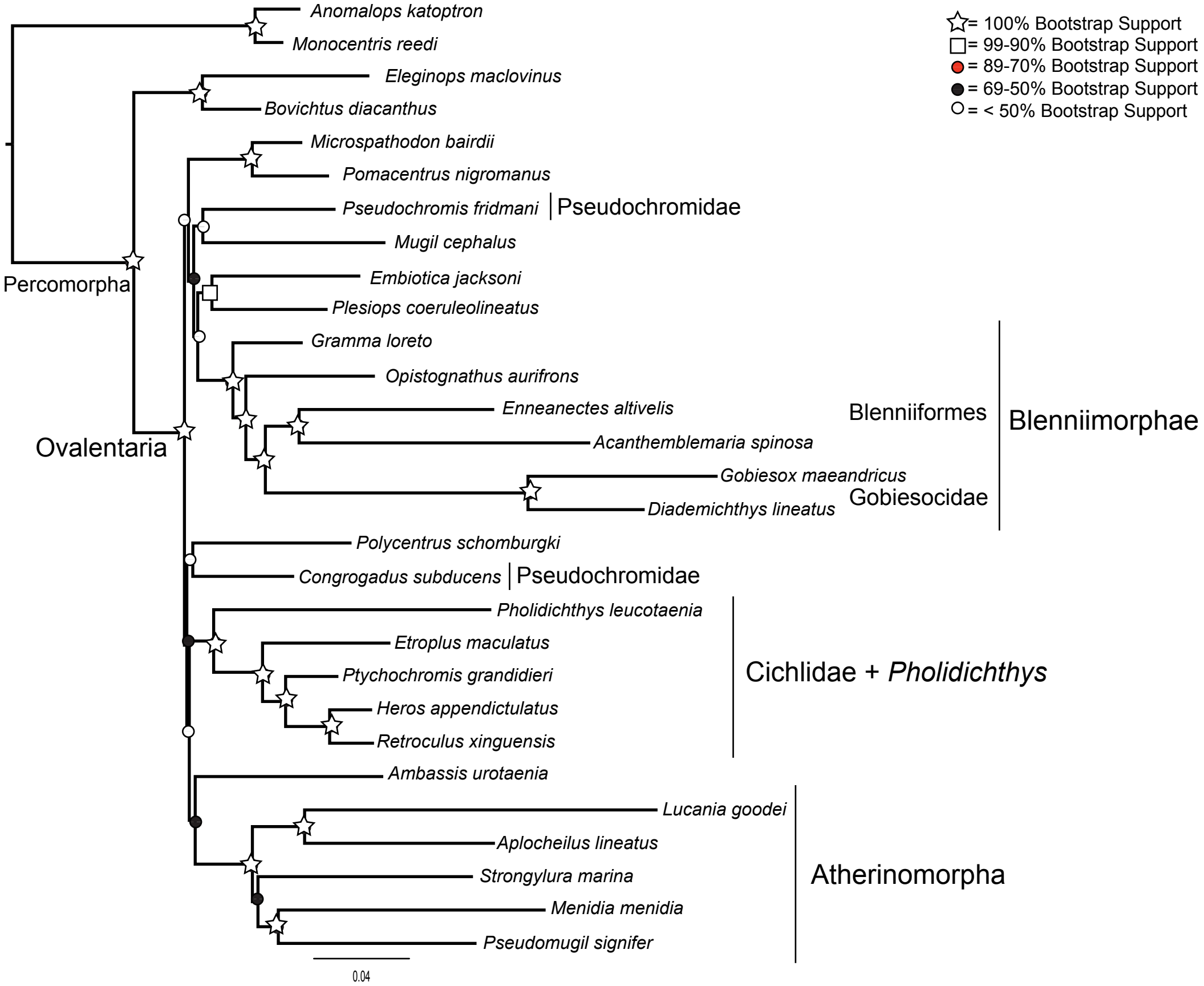

Supplement: Additional file 3: Table S3. — Concatenated maximum likelihood phylogeny inferred using RAxML, from the full 29 species, 107 locus dataset, partitioned by gene. Shapes and colored circles represent bootstrap support for a given node. Higher-level named clades are noted. Note that Pseudochromidae is not a clade. [file 12862_2015_415_MOESM3_ESM.pdf]
