# Supplementary material for: Are 100 enough? Inferring acanthomorph teleost phylogeny using Anchored Hybrid Enrichment
Source: BMC Evol Biol. 2015 Jun 14;15:113. doi: 10.1186/s12862-015-0415-0 (PMC4465735; doi:10.1186/s12862-015-0415-0)
Supplement: Additional file 4: Figure S1. — Concatenated maximum likelihood phylogeny inferred using RAxML, from the full 29 species, 107 locus dataset, inferred using only 3rd codon positions. Shapes and colored circles represent bootstrap support for a given node. Higher-level named clades are noted. Note that Pseudochromidae is not a clade. [file 12862_2015_415_MOESM4_ESM.pdf]

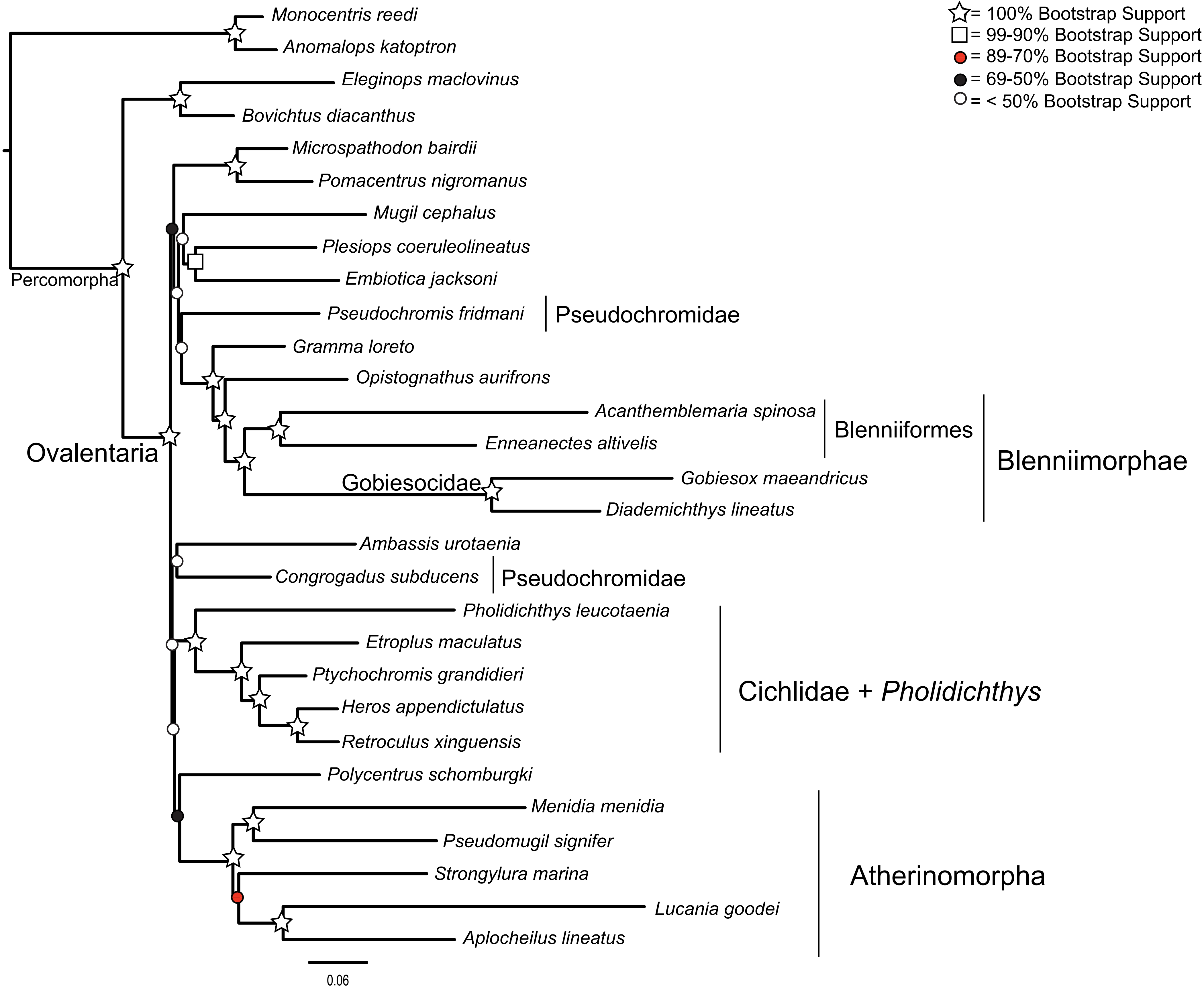

= 100% Bootstrap Support
= 99-90% Bootstrap Support
= 89-70% Bootstrap Support
= 69-50% Bootstrap Support
= < 50% Bootstrap Support
Monocentris reedi
Anomalops katoptron
Eleginops maclovinus
Bovichtus diacanthus
Microspathodon bairdii
Pomacentrus nigromanus
Mugil cephalus
Plesiops coeruleolineatus
Embiotica jacksoni
Pseudochromis fridmani
Pseudochromidae
Gramma loreto
Opistognathus aurifrons
Acanthemblemaria spinosa
Enneanectes altivelis
Blenniiformes
Blenniimorphae
Gobiesocidae
Gobiesox maeandricus
Diademichthys lineatus
Percomorpha
Ovalentaria
Ambassis urotaenia
Congrogadus subducens
Pseudochromidae
Pholidichthys leucotaenia
Etroplus maculatus
Ptychochromis grandidieri
Heros appendictulatus
Retroculus xinguensis
Cichlidae + Pholidichthys
Polycentrus schomburgki
Menidia menidia
Pseudomugil signifer
Strongylura marina
Lucania goodei
Aplocheilus lineatus
Atherinomorpha
0.06
