# Supplementary material for: Are 100 enough? Inferring acanthomorph teleost phylogeny using Anchored Hybrid Enrichment
Source: BMC Evol Biol. 2015 Jun 14;15:113. doi: 10.1186/s12862-015-0415-0 (PMC4465735; doi:10.1186/s12862-015-0415-0)
Supplement: Additional file 5: Figure S2. — Concatenated maximum likelihood phylogeny inferred using RAxML, from the full 29 species, 107 locus dataset, converted to amino acid sequences, under the Dayhoff substitution model. Shapes and colored circles represent bootstrap support for a given node. Higher-level named clades are noted. Note that Pseudochromidae is a clade, albeit with poor support. [file 12862_2015_415_MOESM5_ESM.pdf]

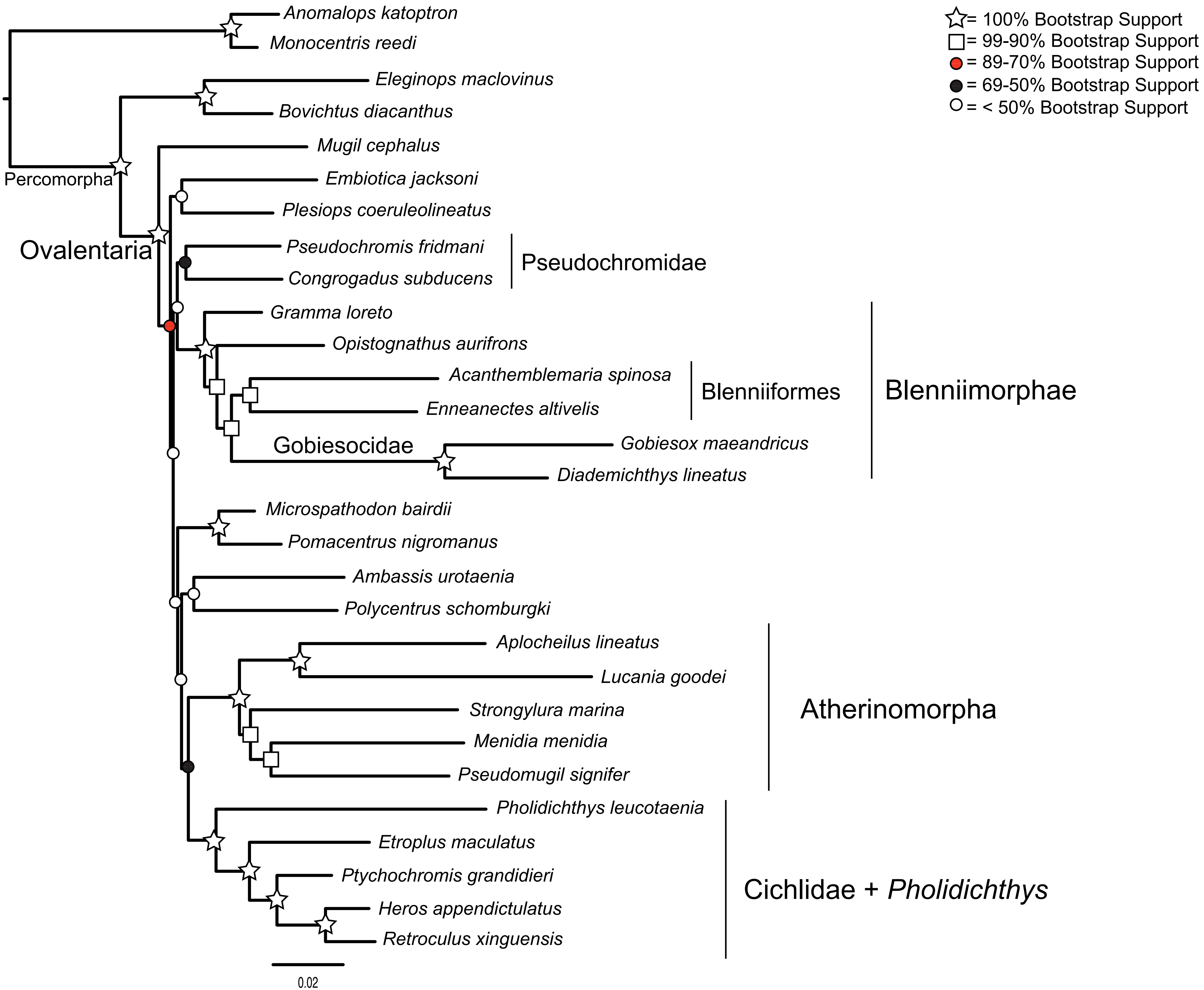
= 100% Bootstrap Support
= 99-90% Bootstrap Support
= 89-70% Bootstrap Support
= 69-50% Bootstrap Support
= < 50% Bootstrap Support
Anomalops katoptron
Monocentris reedi
Eleginops maclovinus
Bovichtus diacanthus
Percomorpha
Mugil cephalus
Embiotica jacksoni
Plesiops coeruleolineatus
Ovalentaria
Pseudochromis fridmani
Congrogadus subducens
Pseudochromidae
Gramma loreto
Opistognathus aurifrons
Acanthemblemaria spinosa
Enneanectes altivelis
Blenniiformes
Blenniimorphae
Gobiesocidae
Gobiesox maeandricus
Diademichthys lineatus
Microspathodon bairdii
Pomacentrus nigromanus
Ambassis urotaenia
Polycentrus schomburgki
Aplocheilus lineatus
Lucania goodei
Strongylura marina
Menidia menidia
Pseudomugil signifer
Atherinomorpha
Pholidichthys leucotaenia
Etroplus maculatus
Ptychochromis grandidieri
Heros appendictulatus
Retroculus xinguensis
Cichlidae + Pholidichthys
0.02
