# Supplementary material for: Are 100 enough? Inferring acanthomorph teleost phylogeny using Anchored Hybrid Enrichment
Source: BMC Evol Biol. 2015 Jun 14;15:113. doi: 10.1186/s12862-015-0415-0 (PMC4465735; doi:10.1186/s12862-015-0415-0)
Supplement: Additional file 6: Figure S3. — Results of cross-validation test. [file 12862_2015_415_MOESM6_ESM.pdf]

	CAT	JTT
	-21501.3	-23252.3
	-21265.9	-23228.4
	-21597.3	-21913
	-21583.9	-22475.9
	-21502.5	-22502.7
	-21057.7	-23020.7
	-21226.2	-23197.8
	-21638.2	-21917.4
	-21762.4	-22371.7
	-21080.1	-22191.3
average	**-21421.6**	**-22607.1**
stddev	245.9986	530.8447
