# Supplementary material for: Are 100 enough? Inferring acanthomorph teleost phylogeny using Anchored Hybrid Enrichment
Source: BMC Evol Biol. 2015 Jun 14;15:113. doi: 10.1186/s12862-015-0415-0 (PMC4465735; doi:10.1186/s12862-015-0415-0)
Supplement: Additional file 7: Table S4. — Species removed for reduced taxon sampling analysis. Available online. [file 12862_2015_415_MOESM7_ESM.pdf]

FAMILY	SPECIES
Aplocheiliidae	Aplocheilus lineatus
Cichlidae	Etroplus maculatus
Cichlidae	Heros appendictulatus
Cichlidae	Ptychochromis grandidieri
Gobiesocidae	Diademichthys_lineatus
Pomacentridae	Pomacentrus nigromanus
Pseudomugilidae	Pseudomugil signifer
Tripterygiidae	Enneanectes altivelis
